# Effects of Switching on the 2-DEG Channel in Commercial E-Mode GaN-on-Si HEMT

**DOI:** 10.3390/mi16101173

**Published:** 2025-10-16

**Authors:** Roberto Baca-Arroyo

**Affiliations:** Department of Electronics, National Polytechnic Institute, School of Mechanical and Electrical Engineering, Mexico City 07738, Mexico; rbaca02006@yahoo.com.mx; Tel.: +52-01-5729-6000

**Keywords:** p-GaN gate HEMT, p-GaN/n-AlGaN/i-GaN heterojunction, low-power GaN transistor, switching conduction mode, didactic physics-based model

## Abstract

In this study, the effects of switching on the two-dimensional electron gas (2-DEG) channel in an E-mode GaN-on-Si HEMT are investigated using a GS-065-004-1-L device that is commercially available for educational practice. A practical prototype with a reduced number of components is proposed, with empirical concepts used to explain its predictive performance when a coreless transformer is series-connected to the E-mode GaN-on-Si HEMT for switching-mode conduction. Conduction modes arising at the p-GaN/n-AlGaN/i-GaN heterojunction in accordance with specifications from the manufacturer’s datasheet were validated using a didactic physical-based model dependent on semiconductor parameters of gallium nitride (GaN). Test circuit-examined waveforms were analyzed, which confirmed that the switching conduction mode of the 2-DEG channel is dependent on physical parameters such as switching operating frequency, temperature, low-field electron mobility, and space charge capacitance.

## 1. Introduction

High-voltage GaN-on-Si transistors have been in development and the subject of intense research since 2015, when the first high-voltage solution was commercially released. These practical devices are based on a cascade configuration of a low-voltage Si-MOSFET in series connection with a high-voltage gallium nitride (GaN) MIS-HEMT, followed by enhancement-mode (E-mode) devices based on a p-doped GaN gate module for low-voltage GaN power transistors. In 2016, the first fully industrial qualified 600 V E-mode GaN power transistors were released by Panasonic and Infineon [1,2]. However, because GaN-based device technologies have been implemented with a standard silicon process to achieve economic balance, one of the biggest challenges when using GaN devices is their reliability [3].

Empirical methods have been established to understand how the formation of piezoelectric effects dependent on charge distribution can contribute to the presence of the conduction path in GaN HEMTs on a very thin (≤30 nm) sheet—called two-dimensional electron gas (2-DEG) in GaN-based devices—where donor states positively impact performance under high electric field conditions, inducing sheet charges at p-GaN/n-AlGaN and n-AlGaN/i-GaN interfaces [4,5]. Therefore, a simplified behavior model of switching effects on the 2-DEG channel is expected to encourage scholars who are researching switching-mode power circuits based on E-mode GaN-on-Si HEMTs, where mounting techniques are needed for long-term temperature cycles in real-world applications, and gate driver techniques with low impedance and high peak current are recommended for fast switching conduction [6]. To understand the physical behavior of a commercial E-mode GaN-based device, empirical concepts used to explain the performance of a test circuit are a valuable educational resource for undergraduate and graduate students.

It is well known that, although technology computer-aided design (TCAD) software packages for power circuit analysis can be a very useful tool for initial design, they are not a substitute for young professionals’ and experienced engineers’ understanding of the overall performance of GaN device-based circuits, because such packages provided by device manufacturers and researchers are usually commercially private and technologically protected. Consequently, to mitigate the lack of design skills and optimize reliability issues using E-mode GaN-on-Si HEMTs, a typical design process must usually begin with specifications for the circuit application, which can proceed based on previous silicon technology-based designs—including specific components and their tolerances—to achieve the best performance by evaluating some of their key physical parameters from a phenomenological viewpoint [7].

The remainder of this study is organized as follows. In Section 2, we provide general description of the experimental setup to present the effects of switching on the 2-DEG channel. In Section 3, we utilize a physics-based model focused on standard carrier conduction equations of silicon-based semiconductor devices for reducing the workload of parameter’s adjustment, and a test circuit and behavior analysis to examine how switching dependence of 2-DEG channel conduction in the commercial GS-065-004-1-L device occurs, particularly when it is driven at frequencies higher than 250 kHz using a coreless transformer in a test circuit. Conclusions about this work are presented in Section 4.

## 2. Experimental Procedure

**Figure 1:**
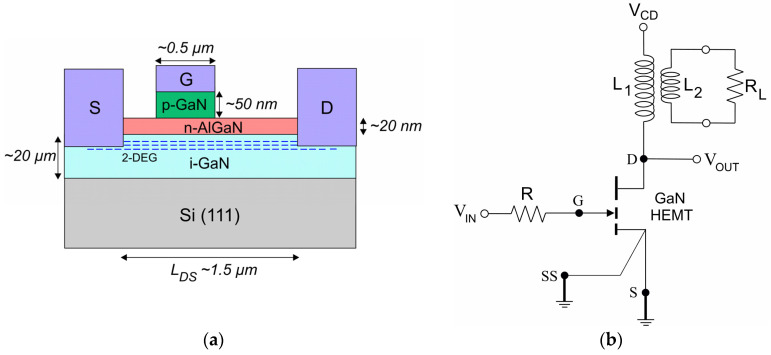
(**a**) Representation of the p-GaN gate structure of the commercial GS-065-004-1-L device; (**b**) schematic diagram of the test circuit for the experimental analysis.

To understand how the effects of switching on the 2-DEG channel behave, an empirical study with accessible measurement instruments for the user is proposed. This study focuses on a physical-based model verified in a test circuit, where a commercial low-power GaN transistor from GaN Systems (Ottawa, ON, Canada) (with part number GS-065-004-1-L) is tested using a range of electrical specifications from the manufacturer’s datasheet. The dimensions for the commercial GS-065-004-1-L device (p-GaN gate structure) shown in Figure 1a comprises an i-GaN buffer, n-AlGaN barrier, p-GaN layer, and three electrodes—namely, drain, source, and gate—where the gate electrode is asymmetrically located at 1/5 *L_DS_* for low-power technology [8,9]. The test circuit in Figure 1b with a reduced number of components is proposed to understand how semiconductor parameters can influence switching effects in the commercial GS-065-004-1-L device.

**Figure 2:**
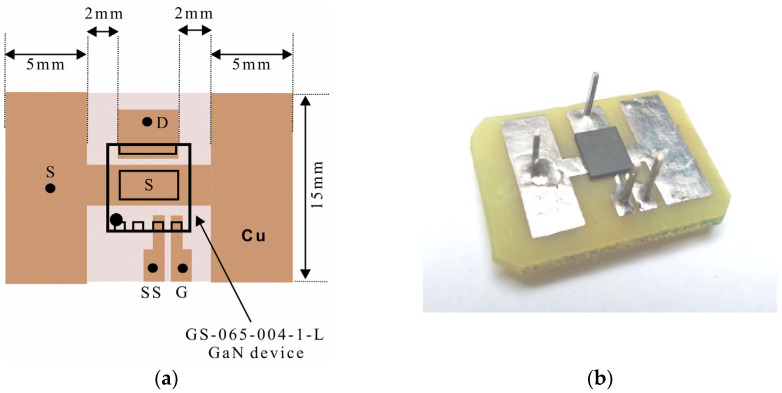
(**a**) PCB layout and related sizes of the mounted GS-065-004-1-L device; (**b**) PCB used to experimentally test it.

Although GaN-based devices behave differently to silicon MOSFETs because they can exhibit an average 2-DEG channel resistance during their turn-off time, they are designed to be cooled using the printed circuit board (PCB), where the source/thermal pad is internally connected [5,10]. Thus, to prevent thermal damage, the copper path on the PCB for distributing the gate drive signal was designed to be as short as possible, while copper paths to the source and drain signals were wider, as shown in Figure 2a. The device was mounted on 0.1 mm PCB thickness phenolic resin as the material, and the copper layer under the source/thermal pad was roughly 15 × 15 mm^2^, as shown in Figure 2b.

## 3. Results and Discussion

### 3.1. Conduction Modes in the E-Mode GaN-on-Si HEMT

Conduction modes for GaN-based devices must be evaluated using semiconductor parameters (e.g., doping concentration, physical dimensions, and electrical characteristics) and well-known physical principles assembled in carrier conduction equations to study its effects with available mathematics software tools. This results in an educational model that could be useful for the predictive analysis of p-GaN/n-AlGaN/i-GaN heterojunctions. To theoretically analyze the conduction modes as a function of the physical parameters, useful specifications from the manufacturer’s datasheet are summarized in Table 1. Furthermore, semiconductor parameters for GaN at room temperature, as listed in Table 2 from data reported in the scientific literature, are taken into account to understand how conduction effects in the practical structure shown in Figure 1b can impact performance in switching-mode power electronics.

Accordingly, to understand how the p-GaN/n-AlGaN heterojunction can affect 2-DEG channel conduction and transiently stimulate the n-AlGaN/i-GaN heterojunction for reliable conduction in an E-mode GaN-on-Si HEMT, standard equations for silicon devices governing static conduction modes can be established as follows.

**Figure 3:**
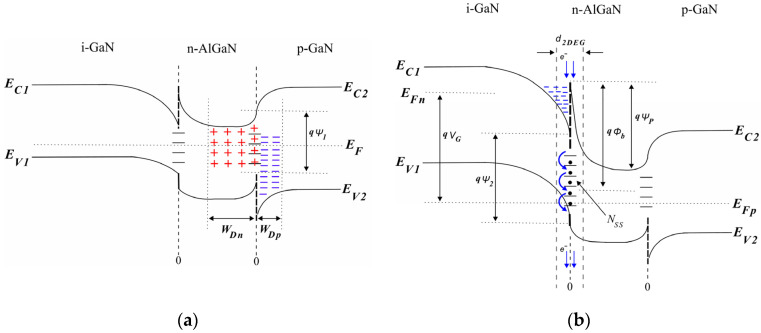
Energy band diagram of the p-GaN/n-AlGaN/i-GaN heterojunction: (**a**) forward bias regime at n-AlGaN/p-GaN heterojunction; (**b**) high-injection regime at i-GaN/n-AlGaN interface under reverse bias condition.

At the p-GaN/n-AlGaN heterojunction, where both sides of the heterojunction become the same material, it is supposed that the diffusion currents are similar to a regular p^+^-n junction and the carrier concentrations are relatively much higher inside the depletion region [12]. Therefore, a dipole layer is supported with the built-in potential, $\Psi_{1}$ and applied bias threshold voltage, where surface effects due to ionic charges can cause the formation of depletion regions $W_{Dp}$ and $W_{Dn}$, giving rise to the high injection level that may occur under a relatively small forward bias condition, as shown in the energy band diagram in Figure 3a. The current/voltage characteristics are established using the following expression:

(1) $$\frac{I_{T}\left(\Psi_{1}\right)}{S_{J1}}=J_{0}\left[e^{\left(\frac{qV_{T}}{kT}\right)}-1\right]$$

where *k =* 8.62 × 10^−5^ eVK^−1^ is the Boltzmann constant; $T=T_{J}+273.15$ in degrees Kelvin, where *T_J_* is the junction temperature in degrees Celsius for the analysis proposed in accordance with the manufacturer’s datasheet; $S_{J1}$ is a cross-section area for mobile carries across the p-GaN/n-AlGaN heterojunction; $V_{T}$ the gate threshold voltage; $J_{0}=qn_{i}^{2}\left[L_{p}{\left(\tau_{p}N_{D}\right)}^{-1}+L_{n}{\left(\tau_{n}N_{A}\right)}^{-1}\right]$ is the saturation current density which depends on $n_{i}$ the intrinsic carrier concentration at room temperature; the carrier lifetime for holes is $\tau_{p}$ and that for electrons is $\tau_{n}$; the diffusion length for holes is $L_{P}\gg W_{Dp}$ and that for electrons is $L_{n}\gg W_{Dn}$; and the impurity concentration for the donor is $N_{D}$ while that for the acceptor is $N_{A}$.

For the n-AlGaN/i-GaN heterojunction, it can be supposed that the conduction mechanism is similar to that governed by thermionic emission where the electron’s conduction regime is influenced by the barrier height, $\phi_{b}$ in which high-mobility electrons can be swept in a velocity saturation regime through the 2-DEG channel length [12,13]. Accordingly, the temperature-influenced current density at the n-AlGaN/i-GaN heterojunction can behave similarly to the metal/semiconductor junction given by

(2) $$\frac{I_{G}\left(\Psi_{2}\right)}{S_{J2}}=\frac{\Psi_{2}A^{*}T_{J}}{k}\left(1-\frac{V_{J}}{\Psi_{2}}\right)e^{\left(-\frac{q\Psi_{2}}{kT_{J}}\right)}e^{\left(-\frac{q\phi_{b}}{kT_{J}}\right)}\left[e^{\left(\frac{qV_{J}}{kT_{J}}\right)}-1\right],$$

where $A^{*}$ = 26.4 × 10^−3^ Acm^−2^K^−2^ is the pre-exponential factor (often expressed in terms of the effective Richardson constant for GaN material [11]), $\Psi_{2}$ is the built-in potential at the i-GaN/n-AlGaN interface, $S_{J2}$ is the cross-section area for mobile electrons across the 2-DEG channel, and $V_{J}$ is the junction voltage at the gate-to-source heterojunction which obeys equation $qV_{J}=q\left(\phi_{b}+\Psi_{p}\right)$ [7,14], where the potential ${\Psi_{p}=qN}^{*}d_{2}^{2}{\left(2K_{GaN}\epsilon_{0}\right)}^{-1}$ is responsible for the trapping reduction and minimizing inactive surface donors at the n-AlGaN barrier of $d_{2}\;\sim \;20\;nm$ (see Figure 1) as a function of the concentration of the ionized donor impurities $N^{*}$ at the i-GaN/n-AlGaN interface, as shown in Figure 3b.

Because the i-GaN buffer is lightly doped, space charge effects independent of the electric field at the n-AlGaN/i-GaN heterojunction can be influenced by $\phi_{b}$, which is sufficient to fully enhance the conduction dominated by the velocity saturation regime. This is due to the presence of free carrier concentrations along the 2-DEG channel length when the gate voltage *V_G_* > *V_T_*, as shown by the energy band diagram in Figure 3b [8,12]. The drain current arising from flow inside the 2-DEG channel can be accurately given by

(3) $$\frac{I_{D}\left(\phi_{b}\right)}{S_{2DEG}}=2\;K_{GaN}\epsilon_{0}v_{S}\frac{V_{D}}{{\left(L+L_{D}\right)}^{2}},$$

where $\epsilon_{0}$ = 8.86 × 10^−14^ Fcm^−1^, $v_{S}$ is the saturation velocity, $S_{2DEG}$ is the cross-section area across the 2-DEG channel length, $L_{D}=\sqrt{{K_{GaN}\epsilon_{0}kT\left(qN^{*}\right)}^{-1}}$ is the Debye length which defines the start-up for conduction limited by space charge effects inside the 2-DEG channel, and *L* = 1/2*L_DS_*, where ${L_{DS}=V}_{DS}F_{BW}^{-1}$ is initially computed using data from Table 1 and Table 2.

Semiconductor parameters for commercial devices are unrevealed by manufacturers and are very hard to determine. However, for the empirical adjustment of Equations (1)–(3), it is sufficient to know initial values of the manufacturer’s data summarized in Table 1 for the extraction of physical parameters $J_{0}$, $\Psi_{2}$, $\phi_{b}$, and $N^{*}$ to accurately reflect the characteristics of the commercial GS-065-004-1-L device. The values of the remaining physical parameters collected in Table 2 are adjusted in accordance with those reported in the scientific literature [10,11].

Based on the energy band diagram of Figure 3 for the p-GaN/n-AlGaN/i-GaN heterojunction, it is pertinent to understand how the junction temperature disturbs the threshold current, *I_T_*, as a function of forward threshold bias *V_T_* ( $\Psi_{1}$), as shown in Figure 3a, as well as gate current *I_G_*, as a function of reverse bias voltage *V_J_* ( $\Psi_{2}$), together with I_D_ ( $\phi_{b}$) at the i-GaN/n-AlGaN interface, as shown in Figure 3b. Thus, because the maximum junction temperature for the commercial GS-065-004-1-L device is 150 °C, current/voltage curves are examined for three temperature values: 40 °C, 80 °C, and 120 °C.

**Figure 4:**
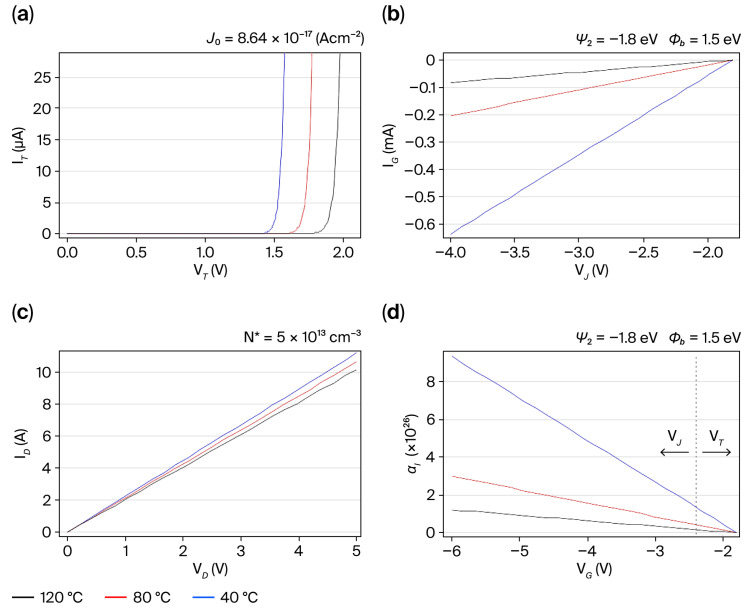
Current/voltage characteristics as a function of the junction temperature and specifications from Table 1: (**a**) *I_T_*–*V_T_* curves; (**b**) *I_G_*–*V_J_* curves; (**c**) *I_D_*–*V_D_* curves; (**d**) $\alpha_{I}$–*V_G_* curves.

The characteristics (*I_T_*–*V_T_*) depicted in Figure 4a show an initial bias current flow at the n-AlGaN/p-GaN heterojunction as a function of the built-in potential $\Psi_{1}$, which is dependent on V_T_ from 1.1 to 2.6 V, as declared in Table 1. In Figure 4b, for the characteristics (*I_G_*-*V_J_*) under reverse bias at the i-GaN/n-AlGaN heterojunction, the current flow begins to increase in the gate-to-source region as a function of $\Psi_{P}$ in *V_J_* ranging from −4 to −2 V when the drain current starts to flow across the drain-to-source distance, where the 2-DEG channel is created at a high injection level, as shown in the characteristics (*I_D_*-*V_D_*) presented in Figure 4c and governed by $\phi_{b}$ and the built-in potential, $\Psi_{2}$, dependent on *V_D_* from 0 to 5 V. Nevertheless, to determine how the switching conduction regime in the commercial GS-065-004-1-L device must be assisted, the evaluation of an injection level coefficient, $\alpha_{I}=I_{G}I_{T}^{-1}$, as presented in Figure 4d, shows how dynamic conduction regime in the p-GaN/n-AlGaN/i-GaN heterojunction is dependent on the gate voltage, V_G_, as a pulse pattern applied in the gate electrode where, to achieve an enhanced injection level at the temperature lower than 80 °C, the *V_T_*/*V_G_* ratio must be lower than 0.3.

Because it is assumed that electrons travel near to the saturation velocity, transit time effects can enable the switching conduction mode through the dipole charge sheet (inside the 2-DEG channel) where current flow can lag behind the voltage based on the time-variant distribution of the electron’s density with respect to the ionized donor impurities’ concentration, $N^{*}$, similar to the space charge-limited conduction (SCLC) formalism for the commercial D-mode GaN-on-Si HEMT [7,12]. Therefore, the trapping of electrons occurs close to the n-AlGaN surface when they are displaced from the depleted i-GaN buffer at the i-GaN/n-AlGaN interface, as shown in the energy band diagram of Figure 3, exhibiting capacitance/frequency characteristics at the drain-to-source heterojunction through the gate width (L_G_~0.5 µm), establishing a connection between $Q_{SS}$ and $\Psi_{P}$ according to ${C_{2DEG}=dQ}_{SS}/d\Psi_{P}$, assuming ${{dQ}_{SS}=I}_{D}(\phi_{b})dt$. Substituting Equation (3) with ${L\approx L}_{G}$ and $dt\approx f_{SW}^{-1}$, taking into account that ${2LL_{D}\gg L}^{2}+L_{D}^{2}$, we can define a space charge capacitance equivalent to the depletion capacitance per unit area, given by

(4) $$C_{D}\approx \frac{K_{GaN}\varepsilon_{0}}{L_{D}L_{G}}\left(\frac{v_{d}}{f_{SW}}\right),$$

where $f_{SW}$ is the switching frequency; $v_{d}\propto \mu_{2DEG}F_{L}$ is the drift velocity (average sound velocity), being proportional to the practical 2-DEG electron mobility, $\mu_{2DEG}$, which is highly dependent on a lateral electric field $F_{L}$ inside the gate-to-drain space, and caused by longitudinal and transverse strains at i-GaN buffer and scattering mechanisms at the n-AlGaN barrier [5,10,11].

Furthermore, the progressive reduction in $N^{*}$ at the n-AlGaN/i-GaN interface and scattering mechanisms along the 2-DEG channel length can slightly impact electron mobility when the temperature rises, as shown in Figure 4c. This is because diffusion mechanisms in the drain-to-source space can occur due to the saturation of the surface state density, and the dependence of $N_{SS}$ on $\Psi_{P}$ rises during 2-DEG channel conduction.

### 3.2. Switching Dependence of Coreless Transformer

It is well known that the output power capability delivered by the coreless transformer to the load for square wave signal excitation [15] can be approximately defined from the stored energy density in the coreless transformer as follows:

(5) $$P_{OUT}=0.5A_{E}^{2}{dB}_{air}f_{sw}10^{-6},$$

where $A_{E}$ is the core effective area in cm^2^; *B_air_* is the air gap flux density; $f_{sw}$ is the switching frequency; and $d=1.5\;A{mm}^{-2}$ is the current density of wires for low-power transformer windings operating at high frequencies, where surface eddy currents are supposed to reinforce the main current flow but oppose it toward the center of the winding [16]. As a result, the induced voltage at the secondary winding of the number of turns, $N_{2}$, could increase as $f_{sw}$ rises, being highly dependent on the primary inductance, L_1_, empirically given by

(6) $$L_{1}=\frac{n}{2I_{2}f_{SW}}\left(DV_{CD}\right),$$

where ${{n=N}_{1}N_{2}^{-1}=I}_{2}I_{1}^{-1}$ is the turn’s ratio and *D* is the duty cycle, defined as the turn-on time/full wave time ratio of a square wave signal with voltage supply $V_{CD}$ and average current flow $I_{2}$ from the secondary winding.

Applying a pulse pattern of a short turn-on time at the gate, as deduced in Figure 4d, the power density transferred to the resistive load from the coreless transformer in Figure 1b could be improved [16,17]. Accordingly, as almost all magnetic energy is stored in air gaps and insulation between conductors, the current flowing around the windings’ surface must be taken into account in terms of skin effects to determine the distribution of B_air_ as a function of its depth, $\delta =\sqrt{{\left(\pi \mu_{0}\sigma_{C}f_{SW}\right)}^{-1}}$, using an expression defined according to the empirical Faraday’s law and $\delta (f_{SW})$ definition, given by

(7) $$\frac{\left(DV_{CD}\right)10^{8}}{2B_{air}N_{1}A_{E}}=\frac{1}{\pi \mu_{0}\sigma_{C}\delta^{2}},$$

where $N_{1}$ is the number of turns for the primary winding of the coreless transformer, $\;A_{E}$ is the core’s effective area in cm^2^, $\mu_{0}=4\pi \;\times \;10^{-7}Hym^{-1}$ is the permeability of vacuum, and ${\sigma_{C}=5.82\times 10}^{7\;}Sm^{-1}$ is the conductivity of the copper wire of the windings.

**Figure 5:**
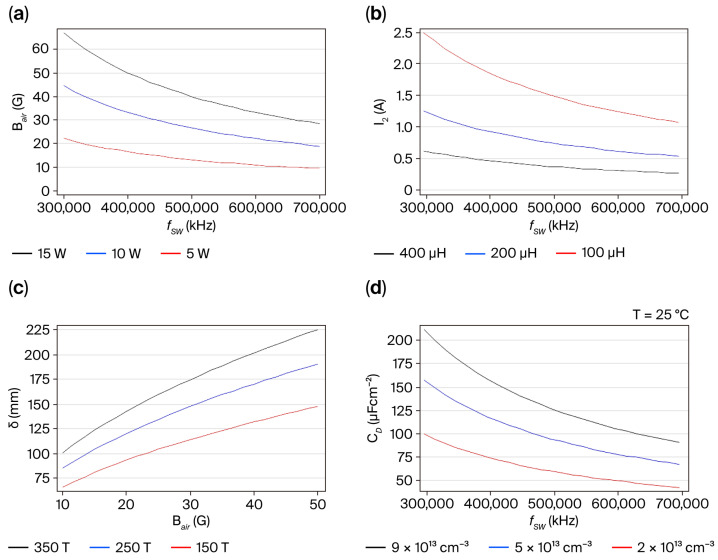
Predictive analysis of the coreless transformer performance at high-frequency switching conduction: (**a**) *B_air_*–*f_SW_* curves; (**b**) *I*_2_–*f_SW_* curves; (**c**) *δ*–*B_air_* curves; (**d**) capacitance/frequency characteristics for commercial GS-065-004-1-L device.

To achieve good power transfer from the input to the load and to circumvent power losses, optimized analysis has been commonly supported using TCAD software packages [14,18]. Nevertheless, instead of using these powerful computer programs for behavior analysis, from empirical Equations (5)–(7), governing the coreless transformer performance can be useful in manufacturing it, because power transfer from a phenomenological viewpoint as a function of physical parameters can be realized in specifications for a practical design, including dimensions, the number of primary and secondary turns, and their operating capabilities at high switching frequencies when series-connected to the depletion capacitance deduced from Equation (4) to understand how GaN-based devices must be operated in the real world and empirically model the drain-to-source heterojunction behavior of the commercial GS-065-004-1-L device intended to be operated in low-power electronics applications. Here, in a theoretical design for the test circuit in Figure 1b, operating specifications such as *V_CD_* = 100 V, *D* = 0.3, 100 µH < L_1_ < 400 µH, *A_E_* = 1 cm^2^, and *n* = 5 are assumed to ensure that a coreless transformer could be reliably developed [19]. The results in Figure 5a–c were computed using the MATHEMATICA 5 software to demonstrate the feasibility of the coreless transformer and space charge capacitance as a function of the switching frequency, $f_{SW}$, in the range from 300 to 700 kHz using Equation (4), as shown in Figure 5d at room temperature.

Figure 5a confirms that B_air_ increases when the power capability rises but, when using an $L_{1}$ higher than 200 µH, as shown in Figure 5b, the $I_{2}$ at the secondary winding might decrease at switching frequencies higher than 350 kHz. However, when the skin depth is taken into account, as shown in Figure 5c, where $N_{1}$ is higher than 150 T (turns), the induced voltage at $L_{2}$ inside the coreless transformer in Figure 1b might be creased within an acceptable *B_air_* level. Hence, it is theoretically confirmed that the coreless transformer manufactured using conventional winding techniques may be applied for switching-mode power circuits at high frequencies, although the winding temperature should be lower than 50 °C for copper wires [18,19]. The depletion capacitance C_D_ decreases as switching frequency rises, as shown in Figure 5d, but its variation as a function of the electron density trapped at three different N* concentrations, 2 × 10^13^ cm^3^, 5 × 10^13^ cm^−3^, and 9 × 10^13^ cm^−3^, is examined.

This confirmed that stable operations can be achieved only at a low injection level (N* = 2 × 10^13^ cm^−3^) to ensure an off-state condition at the *V_OUT_* in Figure 1b (though lower than ½ *V_DS_*; see Table 1), and quasi-resonance phenomena, observed with stored magnetic energy at L_1_ and stored electric energy inside the 2-DEG channel, can result in negligible electrical breakdown effects at switching frequencies higher than 300 kHz.

### 3.3. Analysis of Test Circuit

In accordance with the theoretical results in Figure 5, the test circuit in Figure 1b was built and its performance was experimentally evaluated by connecting a pulse pattern of the positive square-wave signal with *D* = 0.25 to V_IN_, where a series resistor R = 10 Ω was connected on the gate electrode. A coreless transformer series-connected to the drain electrode was built whose specifications are *N*_1_ = 200 T (L_1_~250 µH) of 26 AWG wire, *N*_2_ = 40 T of 22 AWG wire, *A_E_*~1 cm^2^. The load resistor R_L_ = 26.6 Ω (small incandescent lamp for automobile application) was used. The input voltage, *V_IN_*, and output voltage, *V_OUT_*, waveforms were measured using a digital storage oscilloscope (Tektronix (Beaverton, OR, USA), TDS1012C 100 MHz) to determine the physical effects of switching conduction on the 2-DEG channel in the commercial GS-065-004-1-L device.

**Figure 6:**
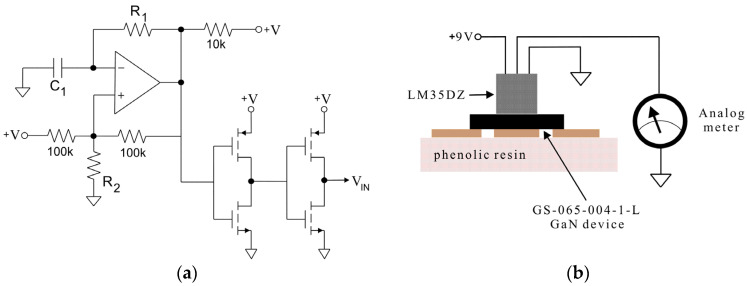
(**a**) Diagram of the driver circuit to build the pulse pattern for switching conduction; (**b**) basic temperature sensing.

A typical driver circuit for the switching performance of GaN devices must comprise one comparator with a programmable turn-on time [20], where the resistor R_2_ can be used to adjust D in the range of 10 to 30%, and two CMOS logic inverter circuits, as shown in Figure 6a, where the capacitor *C*_1_ and resistor R_1_ are both used to adjust the chosen switching frequency. Because the driver circuit used for high-frequency operation requires a bias voltage (*V_GS_* = 6 V) at the gate-to-source heterojunction to ensure stable performance, the blocks specified in Figure 6a are integrated inside the 7555 timer circuit, which was implemented to provide signals, *V_IN_*, in the test circuit (see Figure 1b) for experimental analysis.

The precision integrated-circuit temperature sensor (type LM35DZ; Texas Instruments (Kuala Lumpur, Malaysia)) packaged in TO-92 plastic and designed for a full −55 °C to 150 °C range was chosen to evaluate heat dissipation. The test setup is shown in Figure 6b. It was assembled ensuring that the LM35DZ was as close as possible to the device surface soldered on the PCB and, due to the linear +10 mV per °C scale factor in the LM35DZ, it was easily applied to measure voltage linearly at its output pad using an analog meter.

**Figure 7:**
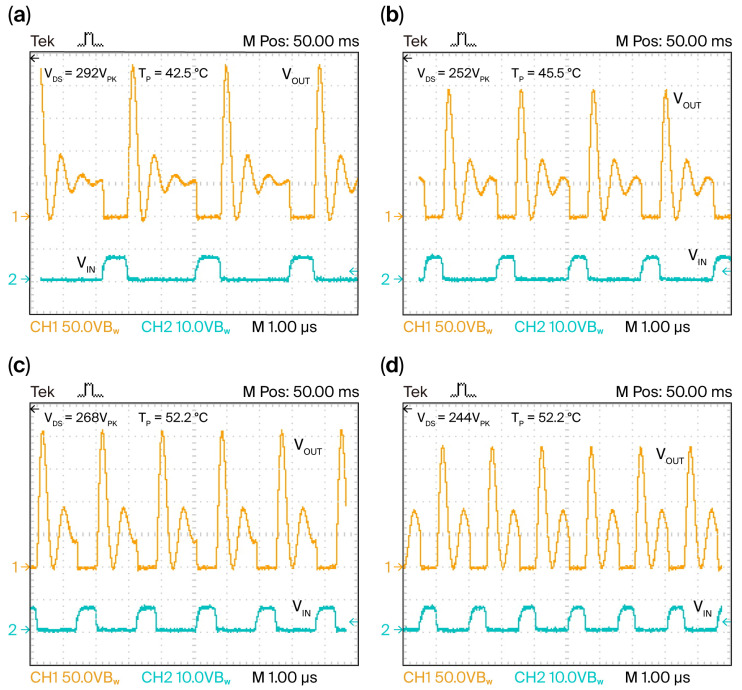
Input and output waveforms under four switching frequencies: (**a**) 350 kHz; (**b**) 450 kHz; (**c**) 550 kHz; (**d**) 650 kHz.

The experimental waveforms displayed in Figure 7a–d show how the commercial GS-065-004-1-L device behaves when the square wave signal with *V_IN_* = 6 V and $f_{SW}$ is in the range of 300 to 700 kHz; moreover, to avoid premature damage, *V_CD_* = 60 V was chosen for the test circuit shown in Figure 1b to satisfy acceptable output power capability, P_OUT_, from Figure 5a and junction temperature, *T_J_*, lower than 80 °C from Figure 4c; therefore, an equivalent series R_ON_-L_1_-C_D_ circuit was identified, where two voltage peaks in the *V_OUT_* signals are observed; the first surge peak is shorter in duration, which is related to the transient behavior of the series R_ON_-L_1_ circuit where stored magnetic energy at L_1_ is fixed on the C_D_ of the drain-to-source heterojunction and the second lower peak of the increasing width in time corresponds to the stored electric energy in the space charge capacitance, C_D_, with 60 V in magnitude. This behaves similarly to the series L_1_-C_D_ quasi-resonant circuit due to the oscillating exchange energy between L_1_ and C_D_ during the off-state, as shown in Figure 7.

The test circuit’s performance results are as follows: At $f_{SW}$ = 350 kHz, an oscillating phenomenon is observed at the second peak with a negligible damping effect, but when $f_{SW}$ = 450 kHz, the oscillating phenomenon increases while the critical damping effect starts to become comparable to the first peak magnitude, resulting in deficient stored magnetic energy at L_1_ and lower induced voltage to the R_L_ from the coreless transformer. Furthermore, the switching conduction mode was observed at $f_{SW}$ = 550 kHz and $f_{SW}$ = 650 kHz, where the oscillating event is more negligible, but there is still stored magnetic energy at L_1_ in the test circuit.

It was observed that the magnitude of the surge peak, V_DS_, decreases as the switching frequency increases, which means that the frequency response of the test circuit verified in Figure 7 is governed by resonance phenomena between L_1_ and C_D_ at the commercial GS-065-004-1-L device and is strongly dependent on the electrical breakdown and strain effects of i-GaN buffer. This is because the turn-off protection (freewheeling diodes and snubber networks) in the test circuit is missing [19,21], but to retain the intention of the reduced number of components, its test circuit was securely operated at V_CD_ < 100 V and the drain-to-source junction operated at *V_DS_* < 300 V, as confirmed in Figure 7.

Because reactivation of flux residues from no-clean soldering paste may cause unwanted conduction paths in GaN devices, scattering mechanisms on the surface of the n-AlGaN barrier under switching conditions as a function of the T_J_ can impact 2-DEG channel conduction [10,22]; therefore, it is aimed to understand how heat dissipation in the source pad on the bottom side of the commercial GS-065-004-1-L device behaves when the device package temperature, T_P_, remains below 100 °C during cooling cycles. The T_P_ was measured as indicated by the experimental results in Figure 7, showing how the temperature dependence of the 2-DEG channel changed when T_P_ increased from 40 to 55 °C and $f_{SW}$ increased, while the damping effects in the wide peak of lower magnitude became negligible, as the oscillating frequency of the equivalent series R_ON_-L_1_-C_D_ circuit began to be equal to the switching frequency of the pulse pattern applied to the gate. This suggests that critical strains due to the lattice and thermal expansion coefficient mismatches at the p-GaN/n-AlGaN/i-GaN heterojunction did not contribute to the presence of the surface donor states at the i-GaN/n-AlGaN interface based on anomalous piezoelectric effects [5,10,14] and the non-uniform distribution of the electric field peaks along the 2-DEG channel length [22].

**Figure 8:**
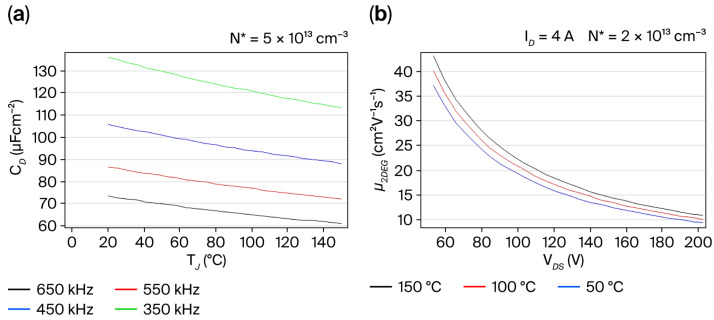
Temperature dependence of conduction mode in 2-DEG channel for E-mode GaN-on-Si HEMT: (**a**) *C_D_*–*T_J_* curves; (**b**) µ_2_*_DEG_*–*V_DS_* curves.

To know how the temperature-dependent dynamic conduction in the 2-DEG channel behaves, a theoretical analysis was evaluated for temperatures ranging from 25 °C to 150 °C, where the commercial GS-0D65-004-1-L device must operate reliably. Figure 8a shows curves describing how the depletion capacitance, $C_{D}\left(T_{J},f_{SW}\right)$, from Equation (4) acts as a function of T_J_ for the four examined switching frequencies, providing useful confirmation of the charge fluctuations in the graphs in Figure 7, where $C_{D}\left(T_{J},f_{SW}\right)$ gradually reduces as T_J_ increases at a medium-ionized impurity level (N* = 5 × 10^13^ cm^−3^). However, low-field electron mobility, ${µ}_{2DEG}\left(V_{DS},T_{J}\right)$, when $v_{S}\;\sim \;v_{d}$ from Equation (3), results in V_DS_ and T_J_ dependence of $\phi_{b}$ = 1.5 eV under a low-ionized impurity level (N* = 2 × 10^13^ cm^−3^), whereas for the three different temperatures, namely, 50 °C, 100 °C, and 150 °C, shown in Figure 8b, it was found that ${µ}_{2DEG}\left(V_{DS},T_{J}\right)$ rapidly reduces as *V_DS_* increases, although it slightly decreases as *T_J_* rises. For the empirical adjustment of Equation (3) describing ${µ}_{2DEG}\left(V_{DS},T_{J}\right)$ as a function of the *V_DS_* and *T_J_*, the average values of $I_{D}=4A$ and ${S_{2DEG}=7.5\times \;10}^{-4\;}{cm}^{2}$ were used to accurately reflect the tendency in the curves of Figure 8b for the commercial GS-065-004-1-L device.

The above-mentioned results indicate that *C_D_* ≤ 100 µFcm^−2^ and $f_{SW}$ ≥ 450 kHz, as well as ${µ}_{2DEG}$ ≥ 10 cm^2^ V^−1^ s^−1^, allowing us to provide a stable conduction mode with a surge V_DS_ peak magnitude lower than 350 V and *T_J_* < 100 °C in accordance with the current/voltage characteristics in Figure 4. The examined results in Figure 7 and predictive analysis in Figure 8 confirm the semiconductor parameters’ dependence of $I_{T}\left(\Psi_{1}\right)$, $I_{G}\left(\Psi_{2}\right)$, $I_{D}\left(\phi_{b}\right)$, $C_{D}\left(T_{J},f_{SW}\right)$, and ${µ}_{2DEG}\left(V_{DS},T_{J}\right)$ in the commercial GS-065-004-1-L device.

Furthermore, the curves in Figure 8 confirm that an ionization level, N*, between 2 × 10^13^ and 5 × 10^13^ cm^−3^ can be responsible for the stable conduction mode of the E-mode GaN-on-Si HEMT when switching conduction in the 2-DEG channel conforms to N*d_2DEG_ >> N_SS_, but its package temperature can increase in a runaway manner above 150 °C generating conductive paths during turn-off time which occur between the drain and Si (111) substrate, as well as between the i-GaN/n-AlGaN interface and Si (111) substrate, which would presumably be responsible for unfavorable transient changes in the occupation of interface states determined by N_SS_, as shown in the energy band diagram in Figure 3b, leading to time-dependent breakdown effects as Si (111) is a material with 10 times lower $F_{BW}$ compared to GaN [10,18,22].

## 4. Conclusions

This research is focused on the effects of switching on the 2-DEG channel of an E-mode GaN-on-Si HEMT to understand how semiconductor parameter-dependent conduction modes can be influenced. Firstly, fine tuning of the current/voltage curves was performed using geometric dimensions ${S_{J1}=1.5\;\times \;10}^{-14}{cm}^{2}$, ${S_{J2}=4.5\;\times \;10}^{-14\;}{cm}^{2}$, and ${S_{2DEG}=7.5\;\times \;10}^{-4\;}{cm}^{2}$. Secondly, a physical parameter-based test circuit was proposed and built within a coreless transformer series-connected to the drain-to-source junction of a commercial GS-065-004-1-L device. Using empirical equations and a simple experiment, reliable performance was demonstrated with a voltage supply lower than 100 V and switching frequencies higher than 300 kHz, validating acceptable heat dissipation in the commercial GS-065-004-1-L device during switching-mode conduction. Based on a didactic physical-based model, this investigation offers a suitable means for behavior analysis and further research to both students and experienced specialists who may face challenges when transitioning to GaN-based devices.

## Figures and Tables

**Table 1:** Specifications for commercial GS-065-004-1-L device.

Parameter	Symbol	Value
Maximum drain-to-source voltage	*V_DS_*	650 V
Gate-to-source threshold	*V_T_*	1.1 to 2.6 V
On resistance	*R_ON_*	0.45 Ω
Gate-to-source current	*I_GS_*	20 µA
Maximum switching frequency	*f_SW_*	~10 MHz
Rise time	*t_R_*	3 ns
Fall time	*t_F_*	11.5 ns
Maximum junction temperature	*T_J_*	150 °C

**Table 2:** Semiconductor parameters for GaN used to evaluate physics-based model [10,11].

Property	Symbol	Value
Bandgap	*E_G_*	3.4 eV
Breakdown field	*F_BW_*	~4 × 10^6^ V/cm
Lateral electric field at drift conduction	*F_L_*	~1.5 × 10^4^ V/cm
Static dielectric constant	*K_GaN_*	9
Intrinsic concentration	*n_i_*	~3.5 × 10^7^ cm^−3^
Hole lifetime	*τ_p_*	~7 ns
Electron lifetime	*τ_n_*	~2 ns
Diffusion length of holes	*L_p_*	~0.8 µm
Diffusion length of electrons	*L_n_*	~8.5 µm
Saturation velocity	*v_S_*	3 × 10^7^ cm/s
Drift velocity	*v_d_*	≤4.5 × 10^5^ cm/s
Surface state density	*N_SS_*	2 × 10^13^ to 5 × 10^13^ cm^−2^
Low concentration at i-GaN	*N_i_*	~2 × 10^9^ cm^−3^
Donor concentration at n-AlGaN	*N_D_*	~5 × 10^16^ cm^−3^
Acceptor concentration at p-GaN	*N_A_*	~2 × 10^18^ cm^−3^
2-DEG channel thickness	*d*_2_ *_DEG_*	≤10 nm

## Data Availability

All of the data are available in the manuscript. Ultimately, the intention of the author is to encourage scholars to explore new empirical research routes by using similar methodologies to those documented here.
